# Effects of Simulated Microgravity on Wild Type and Marfan hiPSCs-Derived Embryoid Bodies

**DOI:** 10.1007/s12195-021-00680-1

**Published:** 2021-06-07

**Authors:** Paola Spitalieri, Mario Marini, Maria Giovanna Scioli, Michela Murdocca, Giuliana Longo, Augusto Orlandi, Giuseppe Novelli, Federica Sangiuolo

**Affiliations:** 1 Department of Biomedicine and Prevention, University of Rome Tor Vergata, Via Montpellier, 1, 00133 Rome, Italy; 2 Department of Systems Medicine, University of Rome Tor Vergata, Rome, Italy; 3 Center of Space Bio Medicine, Microgravity Experimental Service Laboratory, University of Rome Tor Vergata, Rome, Italy; 4 Neuromed Institute, IRCCS, Pozzilli (IS), Italy

**Keywords:** Human induced Pluripotent Stem Cells (hiPSCs), Embryoid Bodies (EBs), Simulated Microgravity (SMG), Marfan Syndrome (MFS), Stem cell differentiation

## Abstract

**Background:**

Mechanical unloading in microgravity is thought to induce tissue degeneration by various mechanisms, including the inhibition of regenerative stem cell differentiation. In this work, we investigate the effects of microgravity simulation on early lineage commitment of hiPSCs from healthy and Marfan Syndrome (MFS; OMIM #154700) donors, using the embryoid bodies model of tissue differentiation and evaluating their ultra-structural conformation. MFS model involves an anomalous organization of the extracellular matrix for a deficit of fibrillin-1, an essential protein of connective tissue.

**Methods:**

*In vitro* models require the use of embryoid bodies derived from hiPSCs. A DRPM was used to simulate microgravity conditions.

**Results:**

Our data suggest an increase of the stemness of those EBs maintained in SMG condition. EBs are still capable of external migration, but are less likely to distinguish, providing a measure of the remaining progenitor or stem cell populations in the earlier stage. The microgravity response appears to vary between WT and Marfan EBs, presumably as a result of a cell structural component deficiency due to fibrillin-1 protein lack. In fact, MFS EBs show a reduced adaptive capacity to the environment of microgravity that prevented them from reacting and making rapid adjustments, while healthy EBs show stem retention, without any structural changes due to microgravity conditions.

**Conclusion:**

EBs formation specifically mimics stem cell differentiation into embryonic tissues, this process has also significant similarities with adult stem cell-based tissue regeneration. The use of SMG devices for the maintenance of stem cells on regenerative medicine applications is becoming increasingly more feasible.

**Supplementary Information:**

The online version contains supplementary material available at 10.1007/s12195-021-00680-1.

## Introduction

The capability of human induced Pluripotent Stem Cells (hiPSCs) to self-renew into undifferentiated cells and, at the same time, to differentiate into all cell types allows us to use them for studying cell differentiation process also in a microgravity environment, in a way similar to what happens during fetal growth.^33^

A key event called gastrulation occurs after implantation of the embryo into the uterus. During this stage of the mammalian development, the preimplantation embryo inner cell mass (ICM) constitutes three germ layers ectoderm (ecto = outer; such as skin and nerves) mesoderm (meso = middle; such as connective, bone and muscle), and endoderm (endo = inner; such as the gut tube and derivatives; like the lung and liver) and three body axes, head-tail, dorsal‐ventral and left‐right, are established. The role and gravity‐sensing of normal gravity (1*g*) acts as an instructive preparation for the gastrulation.^33^

During *in vitro* spontaneous differentiation, hiPSCs form three-dimensional spherical cell aggregates, known as Embryoid Bodies (EBs).^12^ As EB differentiation continues, the cells follow a reproducible temporal pattern that recapitulates early embryogenesis, although without an organized patterning of tissues and organs.^12^ Over time, EBs increase in cell number and complexity as cells form structures comparable to embryonic germ layers, including a wide variety of cell types, such as, cardiomyocytes, hematopoietic cells, and neurons.^3^,^26^,^32^ Although EBs largely exhibit heterogeneous patterns of differentiated cell types, the three-dimensional structure, including paracrine signaling within the EB microenvironment, enables generation and morphogenesis which yields microtissues that are similar to native tissue structures. Such microtissues are able to repair damaged or diseased tissues in regenerative medicine applications, as well as a model of embryonic development.^4^,^29^ For these reasons, EBs have a broad utility to investigate the effects of mechanical unloading also on adult tissue regenerative processes.

To date, there are few studies on mammalian developmental stages from preimplantation embryos through implantation and gastrulation stages in the relationship between physical force and biological function.^1^,^6^,^7^,^42^ Physical forces could also affect the preimplantation embryo through changes in relative buoyancy of cells and blastocoelic fluid or loss of gravity‐dependent convection of fluids adjacent to our inside the embryo.^33^ Cells exposed to microgravity can indeed be profoundly affected by the physical changes that occur in this unique environment, which include the loss of gravity-dependent convection, negligible hydrodynamic shear, and lack of sedimentation. The spatial relationship of cells is a key factor of all the differentiation phases, as this may modify the induction of stress mechanisms. The effects of microgravity and centrifugal forces in a cell are sustained by the most mass dense microstructures. Hence, there is the possibility that a preimplantation embryo in the uterine tube is able to detect microgravity through its organelles or cytoskeletal entities and consequently susceptible to micro displacements, micro shifts or micro translocations. Below a certain threshold (by threshold we mean the force of Earth’s gravity, thus considered as an experimental baseline) of centrifugal forces, cells could respond by rotational movements to reorient themselves nullifying in this way microgravity effects. Above a sustained centrifugal force (above the threshold), the expression of stress molecules are instead stimulated.^2^,^33^

Based on the above considerations, mechanical unloading in microgravity is thought to induce tissue degeneration by various mechanisms, including inhibition of regenerative stem cell differentiation.

The mechanical demands of elastic connective tissue is fulfilled by microfibrils, a main component of the extracellular matrix (ECM). Fibrillin-1, encoded by *FBN1* gene, is the major component of microfibrils, whose function is to provide a scaffold that imparts specific physical properties to various tissues.^30^ This protein forms threadlike filaments called microfibrill within the extracellular matrix.

Mutations in *FBN1* gene cause the Marfan Syndrome (MFS; OMIM #154700), a multisystemic inherited disorder of connective tissue.^14^,^36^

In this study, we investigated the effects of microgravity simulation on early lineage commitment of human induced Pluripotent Stem Cells (hiPSCs) from healthy (WT) and MFS donors, studying the embryoid bodies (EBs) model of tissue differentiation and their ultra-structural conformation. MFS hiPSCs was derived in our lab from a patient carrying a rare deletion comprising the entire *FBN1* gene.^36^ The use of the MFS disease model is due to the characteristic of the disease, which involves an anomalous organization of the extracellular matrix for a deficit of fibrillin-1, an essential protein of connective tissue, providing a scaffold that imparts specific physical properties to various tissues.

To simulate microgravity condition, a Desktop Random Positioning Machine (DRPM) was used, as a valuable tool to facilitate stand-alone studies and cost-efficient platforms for gravitational research. Several expression markers were studied comparing WT versus Marfan cells and also to deeper characterize the subcellular modifications due to SMG, electronic transmission microscopy (TEM) analysis was used to describe both WT and MFS EBs ultrastructural morphology.

## Materials and Methods

### Generation of Patient-Specific hiPSCs

Healthy donor subject (wild type, WT) and Marfan Syndrome (MFS) patient underwent skin biopsy, following written informed consent.

The project was approved by The Committees on Health Research Ethics of Tor Vergata Hospital (2932/2017) and in accordance with the Declaration of Helsinki. Diagnosis of MFS is based on both Ghent clinical criteria and genetic test revealing a rare FBN1 gene deletion (haploinsufficiency).

Human fibroblasts reprogramming was performed, as previously reported.^37^,^38^ hiPSC colonies were picked 20–25 days post infection on the basis of morphology and expanded by plating on mitomycin C-treated MEFs in hiPSCs medium. Successively, hiPS lines MFS and WT were manually picked, passaged on human embryonic stem cell-qualified Matrigel-coated plates (0.05 mg/mL; BD Biosciences) and cultured under feeder-free condition in mTeSR1 medium (Stem Cell Technologies) with Y-27632 ROCK inhibitor (Stemcell Technologies), maintaining the stability over 20 and more passages. The stemness propriety and karyotype analysis were also verified (data not shown).

### Embryoid Body Formation

For the initiation stage of EB formation, we used the same methods as for the hiPSCs passaging, i.e. mechanical cutting of colonies, followed by a mild enzymatic treatment, in order to obtain EBs uniform in size. In fact, at day 0, all hiPS cell colonies were first mechanically cut, followed by detached from the plate bottom by solution containing 1 mg/mL collagenase type IV and Accutase (Gibco), crushed into small similar clusters and floated in ultra-low attachment dishes (Corning 3473) using mTeSR1 medium. At day 1, the medium was replaced with differentiation medium DMEM-F12 supplemented with 20% FBS, 1% non essential amino acid solution, 1 mM l-glutamine, and 1% penicillin/streptomycin for spontaneous differentiation into the three germ layers.

Floating EBs samples were incubated at 37 °C in an incubator with 5% CO_2_ for 24 h.

At day 2, floating EBs were cultured in ultra-low 24 well plates (Corning) on simulated microgravity (SMG) by Desktop Random Positioning Machine (DRPM by Airbus Defence & Space Dutch Technology) for 120 h (5 days). Controls were maintained in normal gravity (1*g*) under the same condition. Then, both 7-day-old 1*g* EBs (WT/MFS) and 7-day-old-SMG EBs (WT/MFS) were transferred to matrigel-coated plates and further differentiated in adesion under normal 1*g* condition until day 22 using the same differentiation medium.

### Simulated Microgravity (SMG) by Desktop Random Positioning Machine

The influence of the force of gravity on EBs cells was assessed using the desktop RPM system (Airbus Defence and Space Netherlands B.V.).^8^ All experiments were carefully planned according to procedures previously described.^16^,^39^,^41^ The rotating frame of the desktop RPM was placed inside an ordinary cell culture CO2 incubator. The software responsible for controlling the motion of RPM employed a tailored algorithm, which rotated with a random speed in such a way that the mean gravity vector reliably converged to zero over time, and it concurrently reduced fluid motion in the culture flask . The samples were positioned compactly in the center of rotation, in order to avoid artifacts and to minimize centrifugal acceleration.

All cell samples were carefully processed for *in vitro* cultivation. We used 24-well plates sealed with dialysis membrane (Visking Medicell International Ltd, Liverpool Road - London code DTV12000.06.000 MWCO 12/14 KDalton). The dialysis membrane was deposited on the convex liquid meniscus of the medium inside the well, allowing it to be sealed and thus preventing the formation of air bubbles. The nitrocellulose discs were fixed to the support by means of a rubber ring. Cells were exposed in culture to the microgravity environment for a continuous period of 120 h. Control samples (1*g*) were cultured and processed in the same manner, but plates were placed beside the RPM machine so that all samples shared identical culture conditions.

### RT-qPCR Analyses

Total RNAs from cells were extracted by Trizol Reagent (Invitrogen Life Technologies Corporation, Carlsbad, CA, USA) following manufacturer’s instructions. Treatment with DNase I-RNase-free (Ambion, Life Technologies Corporation, Foster City, CA, USA) was used to eliminate genomic DNA contamination from total RNA samples. One μg of RNA was reverse transcribed with the High-Capacity cDNA Archive kit (Life Technologies Corporation, Foster City, CA, USA) and used in RT-qPCR. mRNAs were measured by SYBR Green (Life Technologies Corporation, Foster City, CA, USA) using primers listed in Table 1. The comparative ΔΔCt method was used to quantify relative gene expression levels.

**Table 1:** List of primers used in this study.

Primers	Forward	Reverse
OCT4	AACCTGGAGTTTGTGCCAGGGTTT	TGAACTTCACCTTCCCTCCAACCA
SOX2	AGAAGAGGAGAGAGAAAGAAAGGGAGAGA	GAGAGAGGCAAACTGGAATCAGGATCAAA
AFP	AGCTTGGTGGTGGATGAAAC	CCCTCTTCAGCAAAGCAGAC
α-SMA	CTGTTCCAGCCATCCTTCAT	CGGCTTCATCGTATTCCTGT
NCAM	ATGGAAACTCTATTAAAGTGAACCTG	TAGACCTCATACTCAGCATTCCAGT
CCND1	AATGACCCCGCACGATTTC	TCAGGTTCAGGCCTTGCAC
CDKN1A	AGCGGAACAAGGAGTCAG	CGTTAGTGCCAGGAAAGAC
BAX	TGGAGCTGCAGAGGATGATTG	AGCTGCCACCCGGAAGA
BCL2	GAGGCTGGGATGCCTTTGT	CCAGGTATGCACCCAGAGTGA
Brachyury	GCTTCAAGGAGCTCACCAATGAGA	TTCACCTTCAGCACCGGAAACA
Nestin	CTCTGACCTGTCAGAAGAAT	CCCACTTTCTTCCTCATCTG
GATA4	TCTCAGAAGGCAGAGAGTGTG	GGTTGATGCCGTTCATCTTGT
FBN1	AGCGGAGCCGAGCAGTGG	GCTGCTCCCACTTCAGGC
CDH1	CGTCCTGGGCAGAGTGAATTTT	TCACACCATCTGTGCCCACTTT
ELN	GCAAACCTCTTAAGCCAGTTCC	AGACACTCCTAAGCCACCAACT
FBLN1	CACAGGCACAGTGCACGAATG	TCGGGGATGGTTCGGCATT3
COL12A1	ATCTCTGTTATACGCCGGGG	TGCAATTCCCTCTCTGCAGA
COL8A2	AGCACTCTTCCCTTTCTCCC	CCCACAGATGAACCCCTCTT
5.8S	GCGCTAGCTGCGAGAATTAATGTG	CAAGTGCGTTCGAAGTGTCGATGA

### Ultrastructural and Histomorphometric Study

For transmission electron microscopy, 7-old-day EBs (WT/MFS) were fixed in Karnovsky’s solution, processed and embedded in EPON 812.^25^ Thin sections were stained with 0.1% toluidine blue, and ultrathin sections, counterstained with uranyl acetate and lead citrate, and photographed with H-7100FA Hitachi transmission electron microscope (Japan). In addition, histomorphometric analyses were performed on serial EB semi-thin sections under a light microscope connected to a digital camera (E600 Eclipse, Nikon). In particular, EB size (mm^2^) and the percentage ratio between matrix and cell area for each EB were assessed by using ImageJ program (NHI). The apoptotic rate was evaluated by counting the number of apoptotic figures high power field (HPF; ×400, original magnification) in the overall EB area. For each experimental group 20 EBs were analyzed.

### Immunofluorescence (IF) Staining

For immunofluorescence, cells were fixed in 4% paraformaldehyde and incubated with the appropriate primary antibodies against beta III tubulin (TUJ1), Brachyury (BRA) and alpha feto protein (AFP). Appropriate AlexaFluor 488- or 568-labeled secondary antibodies were incubated for 1 h. The cell nucleus was labeled with Hoechst 33342 (Sigma Aldrich) and examined under a fluorescence microscope. Images were acquired using a Zeiss (Thornwood, NY, USA) Axioplan 2 microscope.

### Statistical Analyses

All values provided for RT-qPCR experiments are from independent experiments and are reported as mean values ± standard deviation (SD). Each cell line (WT and MFS) has been tested in triplicate. Data have been compared using the two-tailed Student’s *t* test, for independent samples.

## Results

### Morphological and Molecular Analyses in EBs Cultured in Normal Gravity (1*g*) and Simulated Microgravity (SMG) Conditions

**Figure 1:**
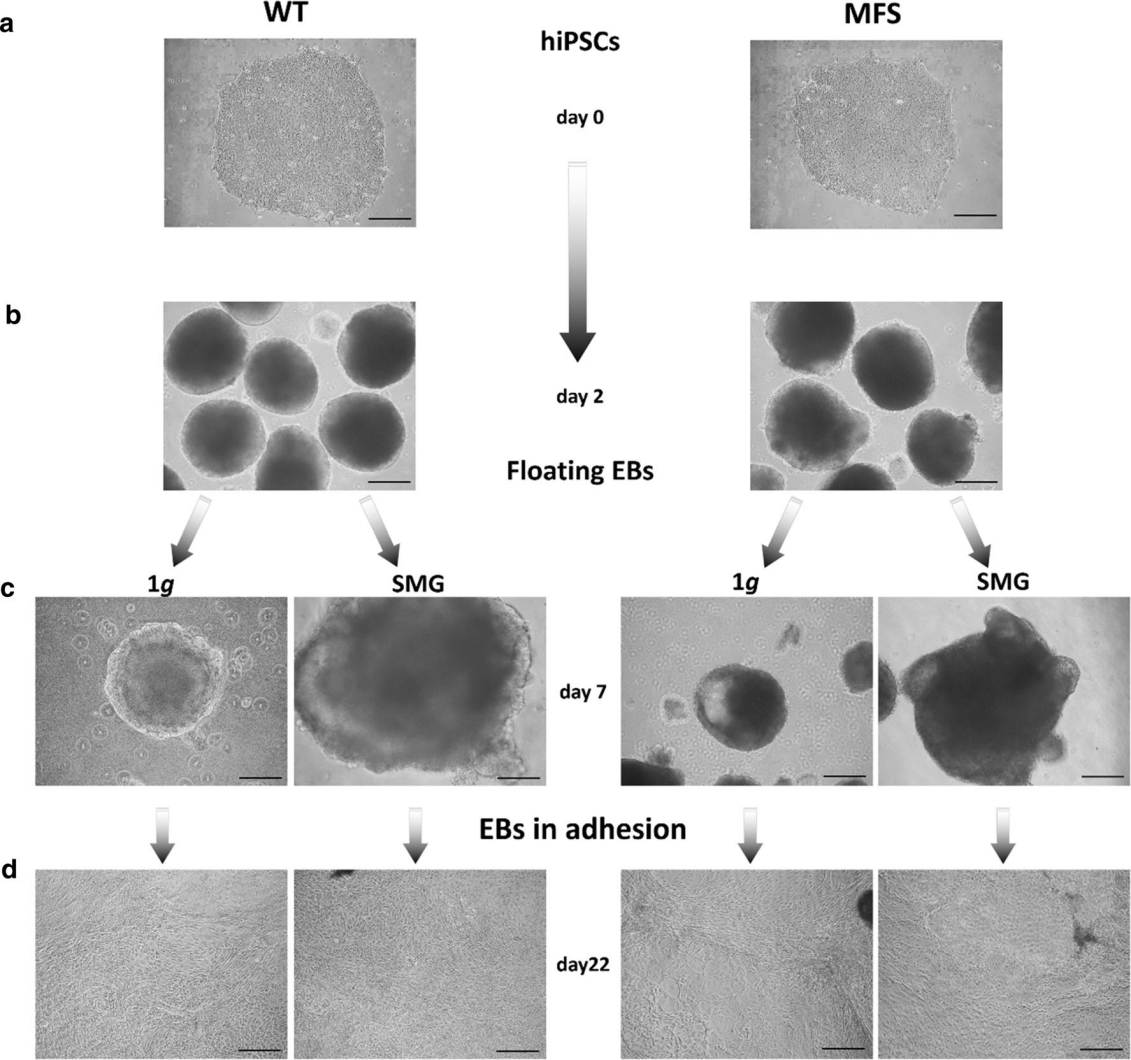
Representative images of EBs WT and MFS during differentation process under 1*g* and SMG conditions. (a) Phase-contrast image representative of hiPS clone WT and MFS in feeder free condition (d 0) and (b) after induction of floating embryoid bodies (EBs) formation at day 2 in normal gravity (1*g*). (c) Floating 7-old-day EBs WT/MFS, in normal gravity (1*g*) and after 120 h in Desktop Random Position Machine (SMG). SMG EBs show a bigger size and in particular MFS an evident morphological irregularities. (d) EBs were spontaneously differentiated under normal 1*g* conditions until day 22. None differences is visible among SMG and 1*g* conditions. Scale bar 20 ×.

Although starting from hiPSCs of comparable size and quality, (Fig. 1a), 2-day-old MFS EBs clearly presented macroscopic morphological irregularities in 1*g* condition respect to WT ones, showing instead a regular round-shape (Fig. 1b).

After 120 h on simulated microgravity (SMG), 7-day-old-SMG EBs (both WT and MFS) showed an increase in size and a less regular form than those remained for the same time in 1*g* condition (control). All EBs also exhibited a thin outer cell layer corresponding to a well-organized differentiation structure, and a cellular thickening in the middle of the body (Fig. 1c). In particular, MFS EBs showed an increase of irregular structures. No further cell suspension or debris due to mechanical stress (i.e. air bubbles) have been observed in SMG plate. Successively, all the EBs were allowed to migrate onto the extracellular matrix (ECM) substrate under normal 1*g* conditions for 15 days, revealing a spontaneous outgrowth. The efficiency of adhesion to tissue cultured treated-plastic of SMG EBs was the same for those maintained in 1*g*, as well as the cell outgrowth surrounding adherent EBs (Fig. 1d). No significant differences were found in the number of EBs that were recovered and adhered on Matrigel in SMG condition compared to 1*g* and between WT and MFS. As expected, only a slight reduction of adhesion was detected on gelatin coating dishes (Online Supplementary Fig. S1**)**. At day 22 of spontaneous differentiation, all cells reached confluence on the culture chamber, ensuring their capacity for outward migration and differentiation into three germ layers, as morphologically evinced by phase–contrast microscope (Fig. 1d) and by immunostaining using markers corresponding to the three germ layers (beta III tubulin—TUJ1, Brachyury—BRA and alpha feto protein—AFP) (Online Supplementary Fig S2) .

**Figure 2:**
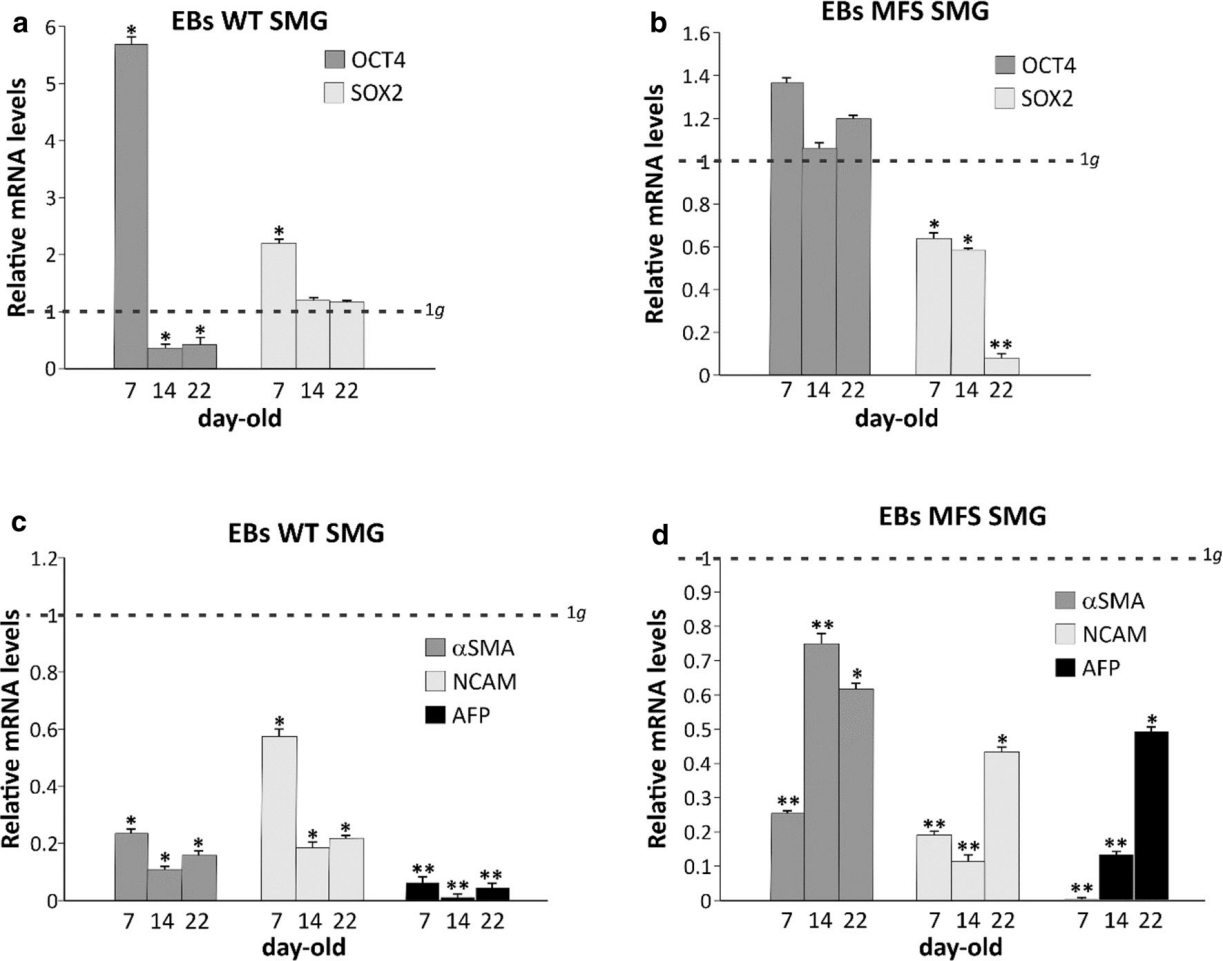
Molecular characterization of stemness and germ layer markers in EBs after 7, 14 and 22 days of spontaneous differentiation. (a) RT-qPCR analyses of OCT4 and SOX-2 transcripts evaluated in WT and in (b) MFS EBs. (c) mRNA expression of the three germ layer markers, alpha smooth muscle Actin (α–SMA), NCAM and α-1-fetoprotein (AFP) by RT-qPCR in WT and in (d) MFS cells. All values are relative to 1*g* condition expressed as unit and represented by a dashed line. 5.8S transcript is used as reference gene. Data are representative of three independent experiments and reported as mean ± SD, (*p value < 0.05, **p value < 0.01).

Molecular analyses have been performed by RT-qPCR to evaluate the expression levels of markers revealing the stemness and the differentiation capabilities of EBs, WT and MFS, at day 7, 14 and 22, comparing among those exposed in SMG and 1*g*. Data analyses have been carried out respect to the expression levels of EBs in 1*g* condition, displayed in the graphs as a dashed line and indicated as unit. The 7-day-old-SMG WT EBs showed a higher expression of both stemness genes OCT4 and SOX2 respect to those in 1*g* (dashed line; *p value < 0.05). At days 14 and 22, SOX2 reached levels comparable to 1*g* condition, while OCT4 strongly decreased (*p value < 0.05) (Fig. 2a). On the contrary, in MFS EBs the expression of OCT4 always remained slightly higher or at least comparable to that in 1*g,* whereas SOX2 showed a downregulation well below the values of EBs cultured in 1*g* condition (*p value < 0.05;**p value < 0.01) (Fig. 2b). Moreover, the gene expression of the three germ layers: mesoderm (αSMA), ectoderm (NCAM) and endoderm (AFP) resulted statistically significant reduced in both WT and MFS (*p value < 0.05; **p value < 0.01) exposed to SMG respect to those in 1*g* (Figs. 2c and 2d). Additionally, three markers were evaluated by RT-qPCR: the transcription factor Brachyury, involved in the development of mesoderm formation and differentiation, and GATA4 and Nestin, endodermal and ectodermal markers respectively. Specifically, Brachyury is thought to regulate genes involved in embryogenesis and in myocardial differentiation as well as GATA 4, while Nestin is a typical neural progenitor. As showed in Supplementary Fig. S3, 7-day-old-SMG WT and MFS EBs exhibited an up-regulation of Brachyury and Nestin respect to those in 1*g* condition (dashed line), while GATA 4 is increased only in 7-day-old-SMG WT EBs (*p value **< **0.05; **p value < 0.01). At day 14 and day 22, all markers were downregulated in both WT and MFS EBs compared to 1*g* (*p value **< **0.05; **p value < 0.01). This trend was slowed down along days because of the prevalence of undifferentiated stem cells in both genotypes.

All values indicated that SMG EBs are less prone to differentiate, providing a measure of the remaining earlier-stage progenitor or stem cell populations. After evaluating these data, it seems that SMG condition slows down the differentiation process, delaying the formation of three germ layers in EBs. This trend is less evident in MFS in which at day 22 the expression of the above markers started to increase, even if not reaching the value of control EBs (1*g* condition) (dashed line) (Fig. 2d).

**Figure 3:**
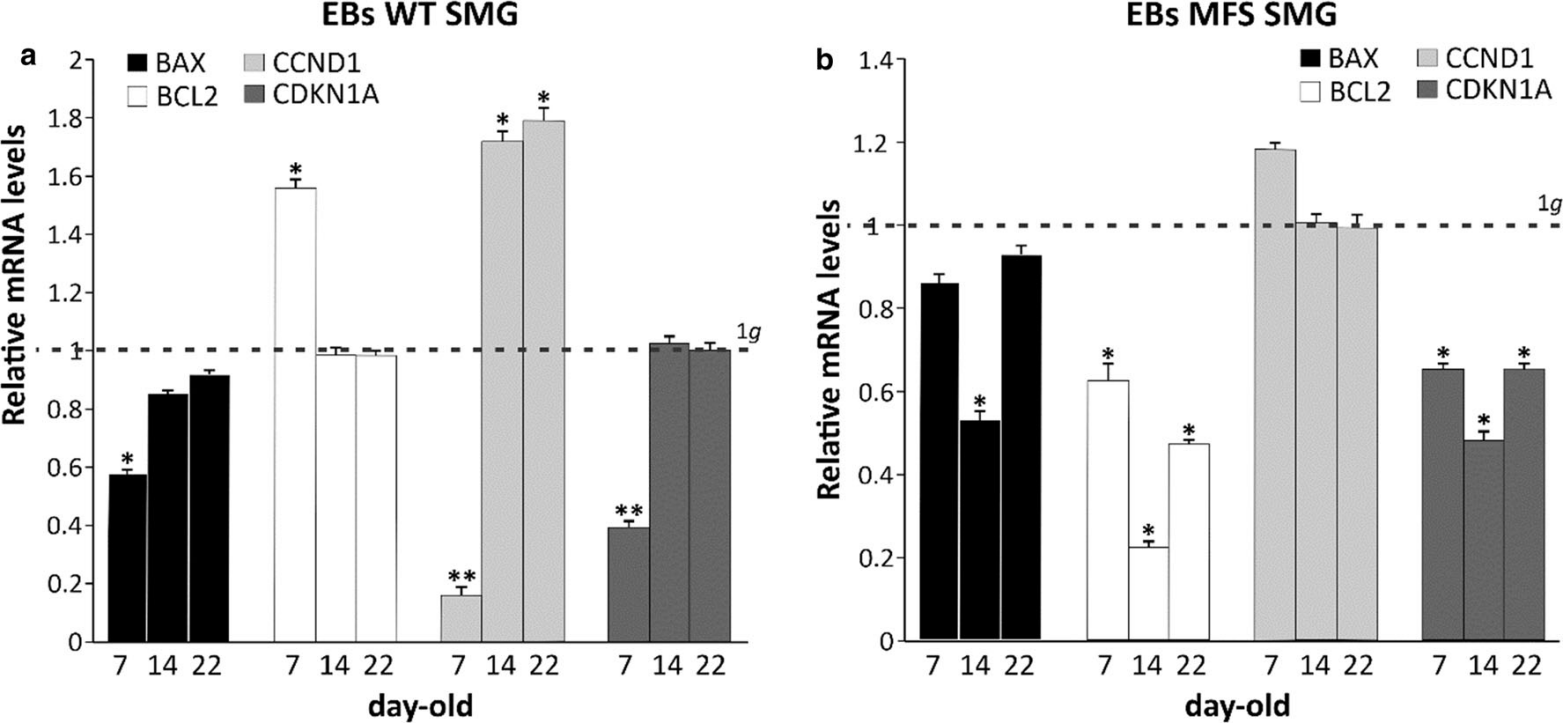
Molecular characterization of cell cycle and apoptosis marker genes in EBs after 7, 14 and 22 days of spontaneous differentiation. (a) RT-qPCR analyses of BAX and BCL2 apoptosis markers and CCND1 and CDKN1A (p21) cell cycle genes in WT and in (b) MFS EBs. All values are relative to 1*g* condition expressed as unit and represented by a dashed line. 5.8S transcript is used as reference gene. Data are representative of three independent experiments and reported as mean ± SD, (*p value < 0.05, **p value < 0.01).

Gene expression of some markers associated with cell cycle regulation and apoptosis-signalling pathway was then investigated, to evaluate any differences due to the exposure to microgravity. The 7-day-old-SMG WT EBs show an inverse relationship among the expression of pro-apoptotic BAX marker and anti-apoptotic BCL2, the latter clearly higher respect to those in 1*g* condition (*p value < 0.05). At day 14 and 22 of differentiation, BAX and BCL2 are equally expressed and matched to values comparable to those in 1*g* (Fig. 3a). On the contrary, in SMG MFS EBs both BAX and BCL2 are never higher than that assessed in 1*g* (*p value < 0.05; Fig. 3b). In the meantime, the expression of CDKN1A gene has been evaluated together with CCND1. CDKN1A encodes for p21 protein, a potent cyclin-dependent kinase inhibitor, able to function as a regulator of cell cycle progression at G1 and S phase DNA replication and damage repair. CCND1 gene encodes the cyclin D1 protein, required for progression through the G1 phase of the cell cycle, regulating cell proliferation, growth, and differentiation. In WT EBs CCND1 expression strongly increases in SMG condition (*p value < 0.05) except for the point at day 7, resulting clearly downregulated (*p value < 0.01; Fig. 3a). On the contrary, in SMG MFS no expression value of CCND1 is significantly higher than those obtained in 1*g* ones. In addition, CDKN1A expression in 7-day-old SMG EBs WT and in 7, 14, 22-day-old MFS EBs is downregulated (*p value < 0.05, **p value < 0.01; Fig. 3b).

Altogether, these data suggested a higher inclination towards stemness in SMG EBs, explaining in this way the delay of their differentiation capacities, as also indicated by a reduced expression of the three germ layer markers, respect to those maintained as controls at 1*g*.

**Figure 4:**
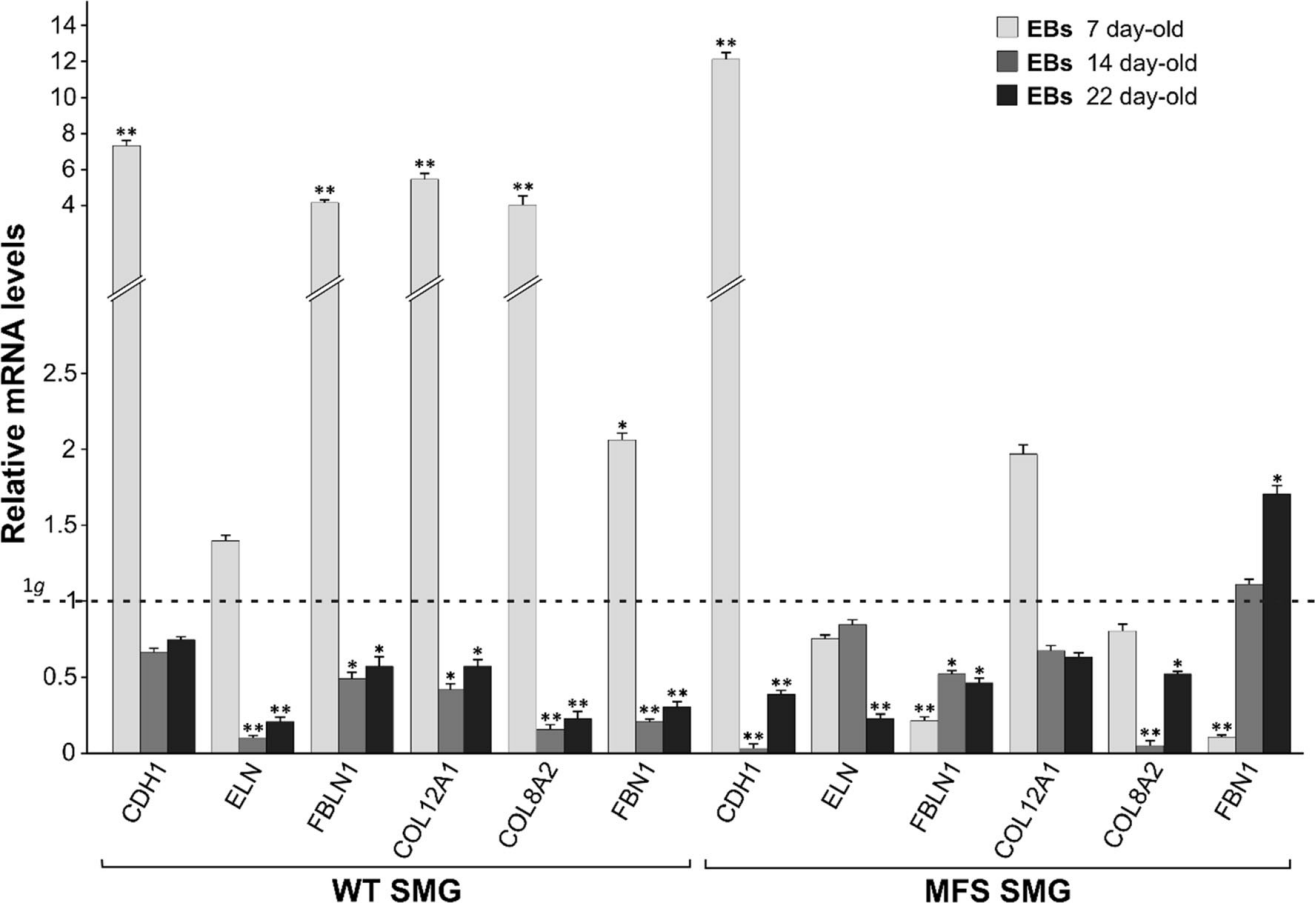
Molecular characterization of extracellular matrix (ECM) marker genes. RT-qPCR analysis of E-Chaderin (CDH1), Elastin (ELN), Fibulin 1 (FBLN1), Collagen Type XII (COL12A1), Collagen Type VIII (COL8A2) and Fibrillin1(FBN1) in floating SMG WT and MFS EBs at 7,14 and 22 days of spontaneous differentiation respect to 1*g* (1*g* expressed as unit; dashed line). 5S is used as reference gene. Data are representative of three independent experiments and reported as mean ± SD (*p value < 0.05, **p value < 0.01).

Finally, we analysed the expression of other important genes that belong to extracellular matrix (ECM), in order to show how other cell structural components were linked to the gravity-dependent effects on the cell differentiation. As FBN1 can polymerize to form microfibrils by interacting with other ECM components including integrins, fibronectins, fibulins, collagens and elastin, we investigated them by Real time-qPCR. In particular, we analysed mRNA expression levels of FBN1, Elastin (ELN), two collagen family genes, such as COL8A2 and COL12A1, and Fibulin (FBLN1), essential for elastic fibre formation. In addition, we examined the expression of E-cadherin (CDH1) that is implicated in mechanisms regulating cell-cell adhesions, mobility and proliferation. As displayed in Fig. 4, 7-day-old-SMG WT EBs exhibited an increase of all markers, in terms of gene expression, compared with 1*g* condition and a decrease along other days (i.e 14 and 22) (*p value **< **0.05; **p value < 0.01). On the contrary, in MFS floating EBs only CDH1 and COL12A1 were up-regulated compared to 1*g* condition at day 7, with a subsequent decrease at day 14 and 22, while all the other markers displayed downregulation (*p value < 0.05; **p value < 0.01). Although MFS EBs on SMG at day 7 are still capable of outward migration as showed by an upregulation of E- cadherin, the expression level of genes involved in ECM organization mirrored the pathological condition characterized by their downregulation. This condition persists also at day 14 and 22. Noterworthy, in MFS-EBs the trend of fibrillin levels is peculiar in SMG condition, in fact FBN expression is significantly reduced at day 7 (**p value < 0.01) while at day 22 a boost in its expression is clearly evident (*p value **< **0.05).

### EB Development is More Irregular in MFS Patient-Derived Cells and Partially Influenced Under SMG Condition

**Figure 5:**
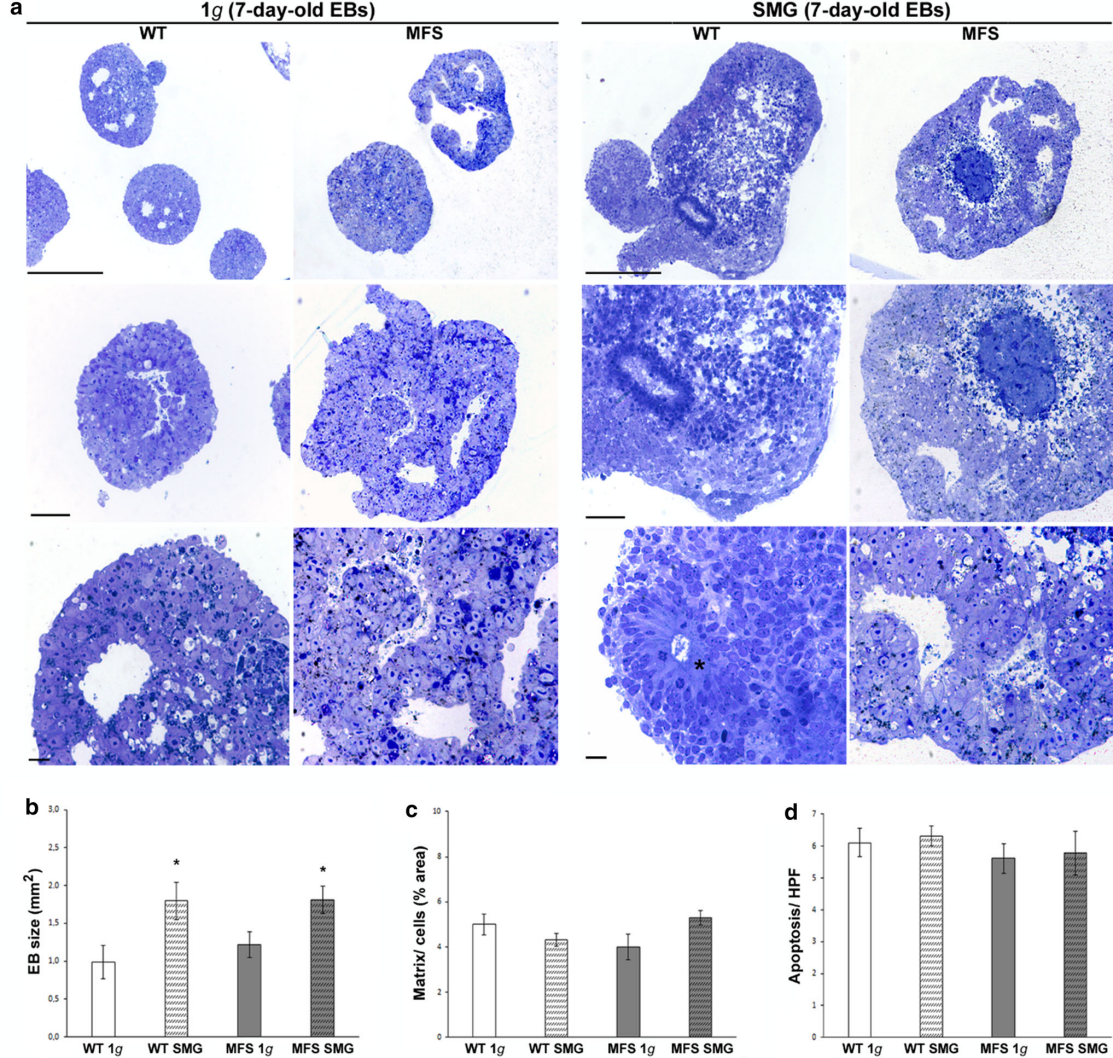
Morphological aspects of WT and MFS EBs under 1*g* and SMG condition. (a) Representative images of toluidine blue-stained semi-thin sections of 7-day-old EBs WT and MFS after 120 h on DRPM (SMG condition) and 1*g* as a control. Most of EBs developed small cavities lined by a columnar epithelium belonging to the inner layer of primitive ectoderm, whereas the outer layer of the EBs consisted of the primitive endoderm-like cells. In some EBs, proliferative neural stem cells, localized in the neural rosettes, are present (asterisk) as well as large areas of necrosis and cellular debris, strongly stained with toluidine blue, filling the cavities. Scale bar = 150 *µ*m. (b–d) Histomorphometric analyses were performed on serial EB semi-thin sections to measure (b) EB size (mm^2^), (c) the percentage ratio matrix/cell area/EB, and (d) the number of apoptotic Fig.s per high power field (HPF) (* *p* value < 0.05).

In both 1*g* and SMG conditions, toluidine blue-stained semi-thin sections of 7-day-old EBs, from WT and MFS hiPS cells, documented small clumps constituted by multilayered cell aggregates (Fig. 5a). In particular, WT cells formed tightly packed EB-like structures; instead, MFS patient-derived cells developed more irregular and disorganized EBs. This situation is more evident in SMG condition, showing large areas of cellular debris, strongly stained by toluidine blue, filled cavities. Interestingly, EB size was greater in SMG than that under 1*g* condition, especially in WT-derived EB (*p value < 0.05; Fig. 5b), which also showed proliferative neural stem cells localized in the neural rosettes (Fig. 5a, asterisk). The percentage ratio between matrix and cell area was similar in WT and MFS EBs under both 1*g* and SMG conditions (Fig. 5c), indicating no changes in matrix secretion and/or accumulation, despite the size enlargement of SMG EBs.

**Figure 6:**
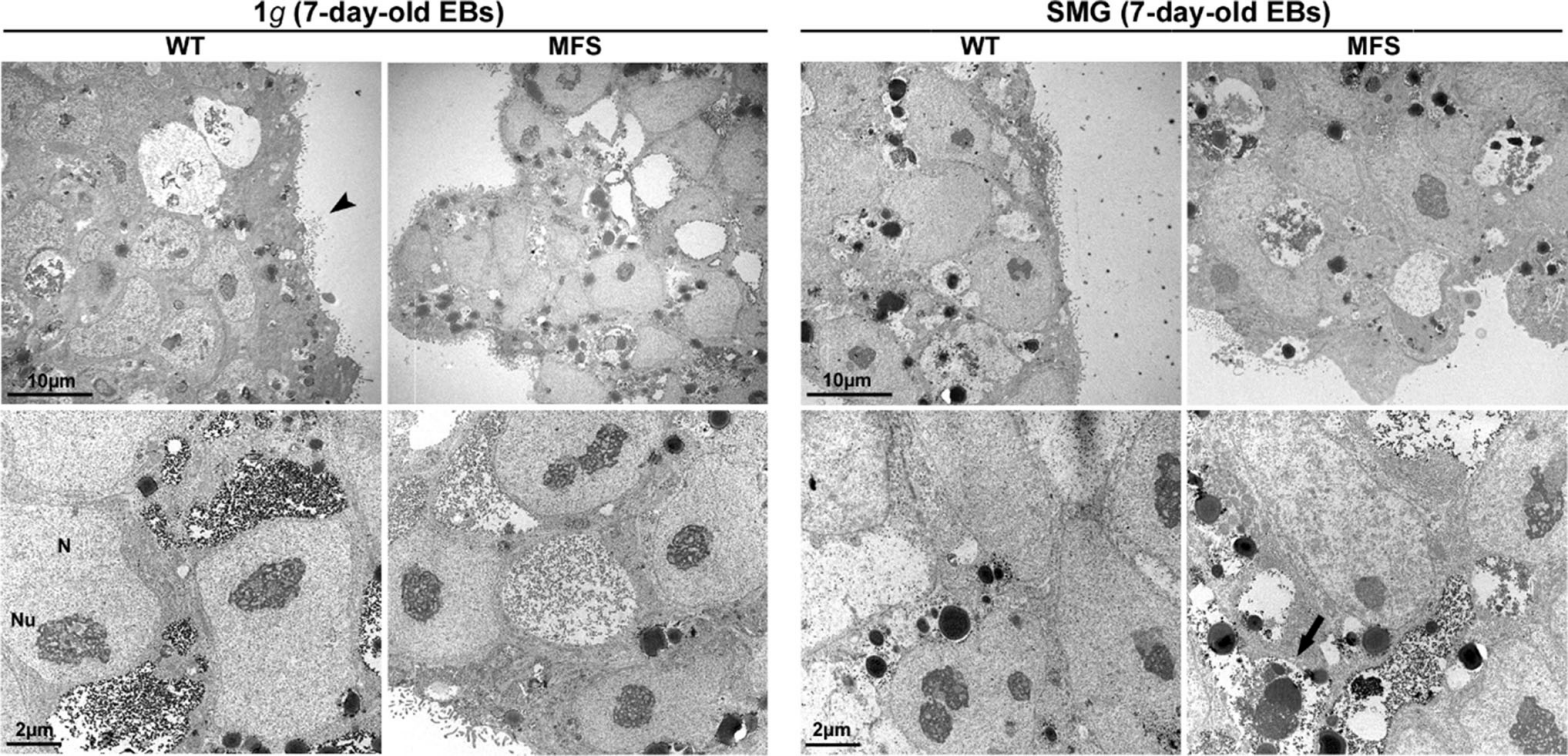
Ultrastructural aspects of WT and MFS EBs under 1*g* and SMG condition. TEM images showing EBs outer layer consisting of the primitive endoderm-like cells with apical microvilli (arrowhead). The nucleus (N) of undifferentiated cells showed dispersed nuclear chromatin with the absence of heterochromatin, prominent and large nucleoli (Nu). In addition, traces of apoptosis and residual cellular fragments are widely present (arrow).

Moreover, the histological morphometric analysis performed on toluidine blue-stained semi-thin sections showed no statistical differences in the number of apoptotic figures per EB total area between all experimental groups (Fig. 5d). TEM investigation revealed that EBs deriving from both WT and MFS developed frequently small cavities lined by a columnar epithelium belonging to the inner layer of primitive ectoderm, whereas the outer layer of EBs consisted mainly of primitive endoderm-like cells with visible apical microvilli (Fig. 6, arrowhead). Ultrastructural analysis also showed the presence of typical undifferentiated cells in EBs with a high nucleus-to-cytoplasm ratio, nuclei with dispersed chromatin and prominent nucleoli, as well as a wide variety of junctions suggesting a complex and specialized structures in both WT and MFS. The physiological process of apoptosis, which characterized EB development, was evident in both WT and MFS, both under 1*g* and SMG conditions (Fig. 6, arrow).

## Discussion and Conclusions

On Earth organisms are constantly subject to contact forces that provide a series of mechanical stimulations essential for the function of many physiological systems. A physiological process of tissue regenerative growth and repair depends on the proliferation and differentiation of tissue-specific stem cells. The lack of gravity and the consequent loss of mechanical stimulation of cells and tissues are essential characteristics of space, which is therefore considered a hostile environment.^10^ In the meantime, the conquest of space like Moon, Mars, /or Spaceflight as well as the emerging technologies more and more often allow astronauts a long stays in conditions of weightlessness and extreme stress conditions with significant health hazards. This requires the study of the effects that these conditions have on the human body and on stem cell-based tissue regenerative processes. However, the limited number of flight opportunities reduced research in the near-earth orbit; for this reason the ground-based simulators of microgravity like DRPM (Desktop Random Positioning Machine) represents valuable tools to facilitate stand-alone studies and cost-efficient platforms for gravitational research and for new technologies for regenerative medicine.^15^,^19^

Recent studies have shown that the use of Random Position Machine (RPM) nullifies the average gravity vector, although over a limited space, by rotating randomly and at variable speeds on multiple axes, mimicking on Earth the conditions of real microgravity present in the space and indicating the state of weightlessness experienced by astronauts inside the spaceship.

The Desktop Random Positioning Machine provides continuous random change in orientation relative to the gravity vector of an accommodated (biological) experiment. When changes in direction are faster than the object’s response time to gravity, DRPM generates effects comparable to those of true microgravity. The DRPM can be operated as a random walk three-dimensional clinostat. The instrument has two independent frames, with a software driving the motors of each frame to random and autonomous but simultaneously smooth and continuous movements. By this way, the location and position of biological samples (like cells) with regard to gravity force vectors are changed quickly and smoothly. This prevents gravity forces from pulling the cells into one direction as in the single-frame Clinostat mode (rotation around one horizontal axis). Finally, an option is provided to generate a file with control parameters that can be reviewed and re-used: a pre-generated random scenario (Moon, Mars, zero gravity). This configuration file, therefore, allows repeating experiments with the same random walk sequence each time.

As already reported, the RPM has been established as a reliable tool supporting ground-based microgravity studies. Effects seen in real microgravity were reproduced with good agreement on RPMs. In order to obtain reliable and comparable data, the appropriate use of the RPM and the application of standardized cultivation methods are of central importance.^39^,^41^

Although the results are sometimes conflicting due to the cellular target or the methodology used (i.e. clinostat or RPM), it is certain the existence of a link between microgravity and impaired tissue renewal by stem cells.^15^

In this study, using the above methodology, we focused to evaluate the effects of simulated microgravity induced by DRPM on human EBs derived from hiPS cells of donors healthy and affected by a monogenic disease (Marfan Syndrome; MFS). The scope was to assess the impact of SMG on embryonic development during the formation of the three primary germ layers. In addition, the use of a disease model based on the deficiency of a structural component of the cytoskeleton, such as fibrillin 1, should allow us to evaluate if the microgravity condition could further exacerbate this scenario.

The results allow us to elaborate countermeasures based solely on understanding physiological changes rather than treating symptoms. We have reported that exposure to SMG condition preserved progenitor stemness and inhibited the expression of differentiation markers for three primary germ layers. Both WT and MFS EBs on SMG are still capable of outward migration, as they become bigger in size before putting them in adhesion. This finding was also confirmed by the histomorphometrical analysis on toluidine blue-stained semi-thin sections that revealed a bigger size of WT and MFS EBs under SMG, whereas a comparable ratio between matrix and cell area under 1*g* and SMG conditions at day 7. The transcription analysis for ECM genes revealed that, elastin, one of the major non-collagenous component of the interstitial ECM,^17^,^35^ did not significantly change in WT and MFS under SMG condition in 7-day old EBs. There was just an early up-regulation of COL8A1 and COL12A1 in WT under SMG condition, but these two molecules only represent minor ECM components that form networks between the different matrix elements by associating to collagen I-containing fibrils.^17^,^35^ So, there is no evidence of a significant alteration in ECM composition in MFS compared with WT EBs. The downregulation of some genes involved in ECM organization and cell-cell adhesion in WT and MFS EBs, along day 14 and 22 under SMG condition, could be probably due to a compensatory phenomenon.

These findings indicate that, despite the EB enlargement in size, no alterations occur in terms of matrix secretion and/or accumulation occur under SMG condition.

The exposure to SMG “freezes” the hiPSC-derived EBs into a pluripotent phenotype, blocking cell differentiation process, as already described in murine stem cells.^6^ In fact, while the stemness genes are more expressed at day 7, the levels of expression relative to the three germ layers, as expected, are reduced in both EBs, even if more evidently in MFS, providing a measure of the remaining earlier-stage progenitor or stem cell populations. In the meantime, 7-day-old SMG EBs were inclined to neural and myocardial differentiation as shown by gene expression of Nestin and Brachyury markers respectively, as expected (Fig. S3).

TEM analysis supported this data showing the presence of proliferative neural stem cells localized in the neural rosettes, especially for WT EBs under SMG condition.

Also, genes associated with the cell cycle and the apoptosis pathway were investigated in order to have an indication about the effects of SMG on their modulation; the pro-apoptotic gene *BAX* and the anti-apoptotic *BCL2* showed respectively inverse expression in WT EBs (downregulation of BAX and upregulation of BCL2), suggesting physiological compensation mechanisms, in order to maintain higher levels of survival. Cell cycle genes are increased in SMG WT respect to 1*g* condition at day 14 and 22, providing indications for a higher stemness condition and a reduced differentiation capability, while in MFS the expression pattern almost never indicated a similar condition. Our data, even if based only on the expression of two markers, suggest in WT EBs an accelerated cell cycle progression in SMG condition at 14 and 22 days, as already described in literature.^6^,^40^ In MFS EBs the apoptosis-related genes are reduced respect at 1*g* condition, with a slight displacement versus the pro-apoptotic response. These data may be also due to normal apoptosis occurring during differentiation inside EBs, when they enlarge and the center of cell masses becomes anoxic and nutrient-deprived, due to lack of vascularization.^21^ This finding is also supported by the ultrastructural analysis that evidenced in MFS patient-derived cells a more irregular EBs formation associated to a size enlargement, with the presence of large areas of strongly toluidine blue-stained and electron dense cellular debris filling cavities, especially under SMG condition. Nevertheless, the histomorphometrical analysis on toluidine blue-stained semi-thin sections revealed a similar apoptotic rate between WT and MFS EBs under both 1*g* and SMG conditions, sustaining the hypothesis of an increased EB size, likely due to a higher stemness under SMG.

Yet, Blaber and collaborators^6^ described as spaceflight preserves the stemness of mouse embryonic stem (ES) cells-derived progenitors and inhibits the expression of markers of terminal differentiation for those tissues derived from the three primary germ layers. Other studies also showed a retention of undifferentiated state of human bone marrow stem cells (BMSC),^9^ or human hepatic tree stem (hBTSCs)^11^ or hematopoietic stem cells, after exposure to SMG.^13^ Results are contradictory, due the different study designs in terms of alternative microgravity simulation (clinorotation vs. Random Position machine) or cell culturing technologies.^15^,^20^,^34^ Anyway, up to date there are few studies on the cellular responses of embryonic stem cells to SMG condition^35^ and none on hiPSC-derived EBs.

Finally, the morphological evaluation suggests in MFS EBs a more pronounced irregularity and disorganization with small outgrowths protruded from larger EBs forming new budding processes. This condition seems to be due to the pathological genotype of EBs rather than to the condition of weightlessness.

It has to be noted that the Marfan genotype of the cells, from which hiPSCs have been derived, carries in heterozygosity a deletion of all coding sequence of *FBN1* gene. This cause a haploinsufficiency of the fibrillin-1 both at transcript and protein level, as already demonstrated.^36^ Molecules of fibrillin-1 bind to each other and to other proteins to form in the extracellular matrix threadlike filaments called microfibrils, components of the elastic fibers and involved in controlling cell growth, proliferation, differentiation, motility, and apoptosis. Previously, some authors have already described a MFS cellular model in which the abnormal FBN1 protein is able to perturb the polymerization of the microfibrils.^31^ Therefore, it is more likely that the decreased FBN1 level could attenuate the extracellular matrix (ECM) integrity.^27^,^28^

Most of the *in vitro* studies on cells indicated that the simulated microgravity influenced cell shape, cytoskeleton, cell migration and growth.^13^ Moreover, while cells cultured under 1*g* conditions have a stretched morphology and undergo to unidirectional migration, cells cultured under SMG undergo to multidirectional migration with directional changes of cell movement. Furthermore, cells cultured under conventional culture conditions maintained their spindle shape through fibronectin fibril formation and focal adhesion, while cells cultured under simulated microgravity resulted partially contracted with fibril structures degraded in the cell bodies and a spatial reorganization of the cytoskeleton.^22^ In the meantime, the adaptive capacity of cells to the microenvironment and their sensitivity to SMG, allows them to quickly make adjustments to the SMG condition. It is possible that, in a condition of fibrillin haploinsufficiency, the adaptive capacity of the cells decreases, as does their migratory capacity.

In particular, it seems that the simulated microgravity favours the expression of fibrillin at day 22 in pathological condition and therefore it could have a positive outcome on the ECM architecture. Another study performed on mesenchymal stem cells reported in microgravity a reduction of the migration capacities together with an evident remodeling the actin cytoskeleton and an increase of cell stiffness.^5^,^24^

Additionally, Lei *et al*. reported that the speed of EB differentiation in space microgravity was slower than at 1*g* condition. Murine EBs have been analyzed on real time during a 15 days spaceflight mission, demonstrating that space microgravity maintains stemness and long-term survival of mESCs, without preventing the migration of EBs on the ECM substrate.^23^ The study also demonstrated that the space microgravity environment might play a potential role in supporting 3D growth of cells and maintenance of stemness in embryonic stem cells.

However, mechanical unloading in microgravity is thought to induce tissue degeneration by various mechanisms, but the retention of more stemness preserves the regenerative process. Due to the observation that multiple stem cell populations show an increased “stemness” and a decreased differentiation capacity under microgravity conditions, the use of microgravity and SMG devices for the maintenance of stem cells for regenerative medicine applications is becoming increasingly more feasible.

Current achievements using RPM technologies for pathological cells represent an important step in the advancement of techniques that may be applied in translational Regenerative Medicine. The field of tissue engineering has achieved great advances in constructing 3D tissues and spheroid or organoid structures from specialized cells or stem cells with and without biomaterials using microgravity. In fact, growing tissues in space and on Earth using microgravity conditions is currently a hot topic in Biomedicine and Regenerative Medicine.^18^

Finally, SMG experiments developed on the RPM are a valid alternative for conducting examinations on the influence of the force of gravity in a fast and straightforward approach. In fact, this could represent the basis for the implementation of the knowledge on microgravity effects on stem cells but also for the study of rare monogenic diseases. Thus, the improvement of SMG-associated cultures could allow the maintaining of vital tissues and organ sections to be potentially destined to clinical therapy.

## Supplementary Information

Below is the link to the electronic supplementary material.

Supplementary material 1 (DOCX 847 kb)
