# Supplementary material for: Effects of Simulated Microgravity on Wild Type and Marfan hiPSCs-Derived Embryoid Bodies
Source: Cell Mol Bioeng. 2021 Jun 7;14(6):613–26. doi: 10.1007/s12195-021-00680-1 (PMC8630351; doi:10.1007/s12195-021-00680-1)
Supplement: Supplementary file 1 — Supplementary material 1 (DOCX 847 kb) [file 12195_2021_680_MOESM1_ESM.docx]

**ON LINE SUPPLEMENTARY FIGURE**

**

**

**FIGURE. S1.** Adhesion on Matrigel or gelatin culture treated dishes of 7-day old EBs WT (A) and MFS (B): Equal number of EBs WT and MFS were plated on 8cm² tissue culture dishes treated with Matrigel or gelatin coating. After 24h, numbers of EBs in adhesion were quantified with light microscopy. No significant differences were found in the number of EBs that were recovered and adhered on Matrigel in SMG condition compared to 1*g* and between WT and MFS. As aspected only a slight reduction of adhesion was detected on gelatin coating dishes (1*g*: Earth’s gravity; SMG: simulated microgravity; Tot EBs SMG: 48; 1*g*: 50).


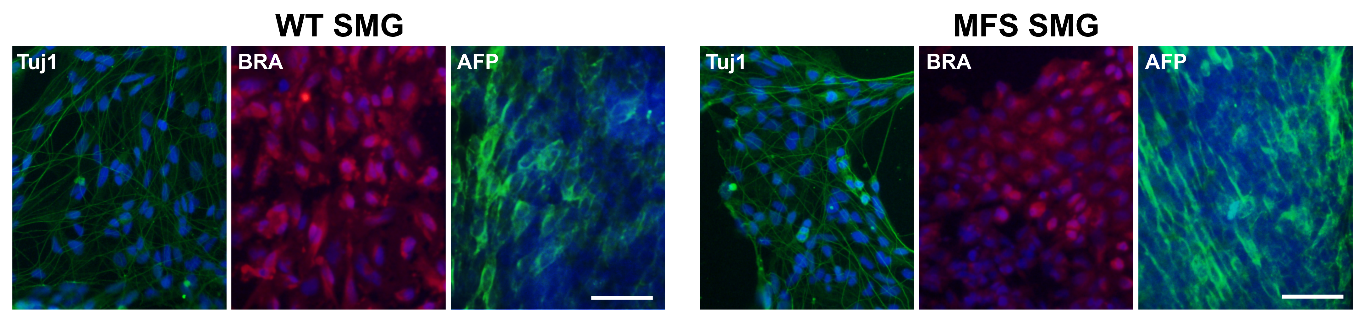


**FIGURE S2**. Images representative of *in vitro* spontaneous differentiation of EBs WT and MFS in adhesion (d22) after SMG condition into the three germ layers. Cells are positive for antibodies specific to early: ectoderm: β-III tubulin (TUJ1, green), mesoderm: Brachyury (Bra; red), and endoderm: α-fetoprotein (AFP; green). Hoechst 33342 nuclear staining in blue. Scale bars = 200µm.





**FIGURE. S3**. Gene expression analyses of mesoderm, ectoderm and endoderm markers precursors. RT-qPCR of Brachyury, Nestin and GATA4 markers in floating EBs at 7,14 and 22 days of SMG respect to those maintained in gravity condition for WT and MFS genotype (1*g* expressed as unit; dashed line). 5S is used as reference gene. Data are representative of three independent experiments and reported as mean±SD (**p* value<0.05, ***p* value<0.01).
